# Aqua­(2,2′-bipyridine-κ^2^ *N*,*N*′){(*E*)-[(5-chloro-2-oxidobenzyl­idene)amino-κ^2^ *N*,*O*]methane­sulfonato-κ*O*}zinc

**DOI:** 10.1107/S1600536812013050

**Published:** 2012-03-31

**Authors:** Yi-Fang Deng, Xue Nie, Chun-Hua Zhang

**Affiliations:** a Hengyang Normal University, Department of Chemistry and Materials Science, Hengyang, Hunan 421008, People’s Republic of China

## Abstract

In the title compound, [Zn(C_8_H_6_ClNO_4_S)(C_10_H_8_N_2_)(H_2_O)], the Zn^II^ atom is six-coordinated by two O atoms and one N atom from a tridentate Schiff base ligand and two N atoms from a chelating 2,2′-bipyridine ligand and one water mol­ecule, forming a slightly distorted octa­hedral geometry. In the crystal, O—H⋯O hydrogen bonds link pairs of complex mol­ecules into dimers. An intra­molecular O—H⋯O hydrogen bond is also present.

## Related literature

For related complexes, see: He *et al.* (2007 ▶); Xu *et al.* (2007 ▶).
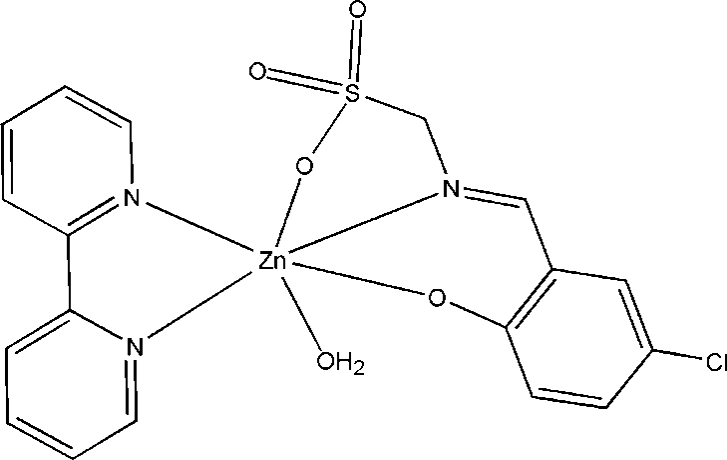


## Experimental

### 

#### Crystal data

[Zn(C_8_H_6_ClNO_4_S)(C_10_H_8_N_2_)(H_2_O)]
*M* *_r_* = 487.22
Triclinic, 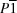

*a* = 7.7332 (6) Å
*b* = 10.8948 (8) Å
*c* = 11.9112 (9) Å
α = 103.625 (1)°
β = 90.664 (2)°
γ = 98.993 (1)°
*V* = 962.08 (13) Å^3^
*Z* = 2
Mo *K*α radiation
μ = 1.56 mm^−1^
*T* = 296 K
0.18 × 0.14 × 0.10 mm

#### Data collection

Bruker APEX CCD diffractometer
Absorption correction: multi-scan (*SADABS*; Sheldrick, 1996 ▶) *T*_min_ = 0.766, *T*_max_ = 0.860
7155 measured reflections
3719 independent reflections
3345 reflections with *I* > 2σ(*I*)
*R*_int_ = 0.026

#### Refinement

*R*[*F* ^2^ > 2σ(*F* ^2^)] = 0.028
*wR*(*F* ^2^) = 0.077
*S* = 1.05
3719 reflections
267 parameters
H atoms treated by a mixture of independent and constrained refinement
Δρ_max_ = 0.27 e Å^−3^
Δρ_min_ = −0.52 e Å^−3^

### 

Data collection: *SMART* (Bruker, 2007 ▶); cell refinement: *SAINT* (Bruker, 2007 ▶); data reduction: *SAINT*; program(s) used to solve structure: *SHELXTL* (Sheldrick, 2008 ▶); program(s) used to refine structure: *SHELXTL*; molecular graphics: *SHELXTL*; software used to prepare material for publication: *SHELXTL*.

## Supplementary Material

Crystal structure: contains datablock(s) global, I. DOI: 10.1107/S1600536812013050/hy2525sup1.cif

Structure factors: contains datablock(s) I. DOI: 10.1107/S1600536812013050/hy2525Isup2.hkl

Additional supplementary materials:  crystallographic information; 3D view; checkCIF report

## Figures and Tables

**Table 1:** Hydrogen-bond geometry (Å, °)

*D*—H⋯*A*	*D*—H	H⋯*A*	*D*⋯*A*	*D*—H⋯*A*
O1*W*—H1*WA*⋯O4^i^	0.84 (3)	1.96 (3)	2.791 (2)	172 (3)
O1*W*—H1*WB*⋯O3	0.82	2.10	2.855 (2)	154

Symmetry code: (i) 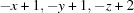.

**Fig. 1.**
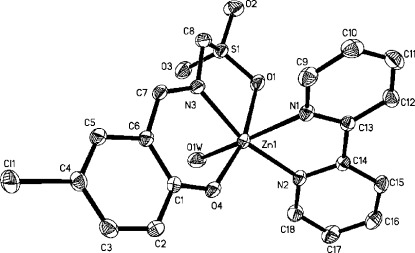
The molecular structure of the title compound. Displacement ellipsoids are drawn at the 30% probability level. H atoms have been omitted for clarity.

## supplementary crystallographic information

### Comment

Schiff base complexes containing amino acids have been studied for many years, arising interest because of their antiviral, anticancer and antibacterial activity. Herein, we choose amino-methanesulfonic acid-Schiff base to react with Zn(CH_3_CO_2_)_2_.4H_2_O as well as 2,2'-bipyridine. In the title compound, the Zn^II^ atom forms one five-membered and one six-membered chelating rings with the Schiff base ligand (Fig. 1). The coordinating environment of the Zn^II^ atom is different from that reported by He *et al.* (2007) and Xu *et al.* (2007). The bond length of Zn—O(sulfonate) is longer than that of Zn—O(aqua) and also longer than that of Zn—N(imine). It indicates that the coordinating capability of the sulfonate group is weaker than that of water and imine group. In the crystal, O—H···O hydrogen bonds link two complex molecules into a dimer (Table 1). An intramolecular O—H···O hydrogen bond is also present.

### Experimental

The complex was prepared by mixing a methanol-water solution of 5-chlorosalicylaldelyde (1.0 mmol), aminomethanesulfonic acid (1.0 mmol) and potassium hydrate (1.0 mmol) with heating and stirring. After 2 h, an aqueous solution containing zinc acetate (1 mmol) was added dropwise under stirring. The pH value of the mixture was adjusted to 6 with 0.5 mol/*L* HCl solution, followed by the dropwise addition of a methanol solution containing 2,2'-bipyridine (1 mmol) with stirring. The resulting yellow filtrate was allowed to stand at room temperature and slowly evaporate for one month to afforded yellow block-shaped crystals.

### Refinement

H atoms attached to C atoms were positioned geometrically and refined as riding atoms, with C—H = 0.93 (CH) and 0.97 (CH_2_) Å and with *U*_iso_(H) = 1.2*U*_eq_(C). The water H atoms were located from a difference Fourier map, one of them was refined isotropically and the other was refind as riding, with *U*_iso_(H) = 1.5*U*_eq_(O).

### Crystal data

**Table:**

[Zn(C_8_H_6_ClNO_4_S)(C_10_H_8_N_2_)(H_2_O)]	*Z* = 2
*M**_r_* = 487.22	*F*(000) = 496
Triclinic, *P*1	*D*_x_ = 1.682 Mg m^−^^3^
Hall symbol: -P 1	Mo *K*α radiation, λ = 0.71073 Å
*a* = 7.7332 (6) Å	Cell parameters from 4928 reflections
*b* = 10.8948 (8) Å	θ = 2.3–27.5°
*c* = 11.9112 (9) Å	µ = 1.56 mm^−^^1^
α = 103.625 (1)°	*T* = 296 K
β = 90.664 (2)°	Block, yellow
γ = 98.993 (1)°	0.18 × 0.14 × 0.10 mm
*V* = 962.08 (13) Å^3^	

### Data collection

**Table:**

Bruker APEX CCD diffractometer	3719 independent reflections
Radiation source: fine-focus sealed tube	3345 reflections with *I* > 2σ(*I*)
Graphite monochromator	*R*_int_ = 0.026
φ and ω scans	θ_max_ = 26.0°, θ_min_ = 1.8°
Absorption correction: multi-scan (*SADABS*; Sheldrick, 1996)	*h* = −5→9
*T*_min_ = 0.766, *T*_max_ = 0.860	*k* = −13→13
7155 measured reflections	*l* = −14→14

### Refinement

**Table:**

Refinement on *F*^2^	Primary atom site location: structure-invariant direct methods
Least-squares matrix: full	Secondary atom site location: difference Fourier map
*R*[*F*^2^ > 2σ(*F*^2^)] = 0.028	Hydrogen site location: inferred from neighbouring sites
*wR*(*F*^2^) = 0.077	H atoms treated by a mixture of independent and constrained refinement
*S* = 1.05	*w* = 1/[σ^2^(*F*_o_^2^) + (0.039*P*)^2^ + 0.2378*P*] where *P* = (*F*_o_^2^ + 2*F*_c_^2^)/3
3719 reflections	(Δ/σ)_max_ = 0.001
267 parameters	Δρ_max_ = 0.27 e Å^−^^3^
0 restraints	Δρ_min_ = −0.52 e Å^−^^3^

### Special details

**Table:**

Geometry. All e.s.d.'s (except the e.s.d. in the dihedral angle between two l.s. planes) are estimated using the full covariance matrix. The cell e.s.d.'s are taken into account individually in the estimation of e.s.d.'s in distances, angles and torsion angles; correlations between e.s.d.'s in cell parameters are only used when they are defined by crystal symmetry. An approximate (isotropic) treatment of cell e.s.d.'s is used for estimating e.s.d.'s involving l.s. planes.
Refinement. Refinement of *F*^2^ against ALL reflections. The weighted *R*-factor *wR* and goodness of fit *S* are based on *F*^2^, conventional *R*-factors *R* are based on *F*, with *F* set to zero for negative *F*^2^. The threshold expression of *F*^2^ > σ(*F*^2^) is used only for calculating *R*-factors(gt) *etc*. and is not relevant to the choice of reflections for refinement. *R*-factors based on *F*^2^ are statistically about twice as large as those based on *F*, and *R*- factors based on ALL data will be even larger.

### Fractional atomic coordinates and isotropic or equivalent isotropic displacement parameters (Å^2^)

**Table:**

	*x*	*y*	*z*	*U*_iso_*/*U*_eq_	
Zn1	0.62092 (3)	0.62060 (2)	0.841029 (19)	0.03292 (9)	
C1	0.8756 (3)	0.68019 (19)	1.04504 (17)	0.0312 (4)	
C2	0.9837 (3)	0.6387 (2)	1.11991 (18)	0.0382 (5)	
H2	0.9938	0.5524	1.1046	0.046*	
C3	1.0741 (3)	0.7212 (2)	1.21429 (18)	0.0382 (5)	
H3	1.1456	0.6911	1.2614	0.046*	
C4	1.0588 (3)	0.8500 (2)	1.23969 (18)	0.0367 (5)	
C5	0.9626 (3)	0.8967 (2)	1.16761 (18)	0.0343 (4)	
H5	0.9573	0.9838	1.1839	0.041*	
C6	0.8707 (2)	0.81353 (18)	1.06824 (17)	0.0298 (4)	
C7	0.7749 (3)	0.87525 (18)	0.99868 (17)	0.0305 (4)	
H7	0.7835	0.9637	1.0229	0.037*	
C8	0.5976 (3)	0.90082 (19)	0.84771 (18)	0.0358 (5)	
H8A	0.6656	0.9161	0.7830	0.043*	
H8B	0.5928	0.9827	0.9007	0.043*	
C9	0.8962 (3)	0.7207 (2)	0.6752 (2)	0.0458 (6)	
H9	0.9395	0.7860	0.7392	0.055*	
C10	0.9547 (3)	0.7293 (2)	0.5682 (2)	0.0515 (6)	
H10	1.0374	0.7980	0.5600	0.062*	
C11	0.8876 (3)	0.6336 (3)	0.4738 (2)	0.0500 (6)	
H11	0.9206	0.6389	0.4001	0.060*	
C12	0.7714 (3)	0.5298 (2)	0.48847 (19)	0.0407 (5)	
H12	0.7273	0.4634	0.4254	0.049*	
C13	0.7217 (2)	0.52624 (19)	0.59897 (17)	0.0298 (4)	
C14	0.6071 (2)	0.41548 (18)	0.62564 (17)	0.0299 (4)	
C15	0.5698 (3)	0.2974 (2)	0.54736 (19)	0.0393 (5)	
H15	0.6097	0.2864	0.4729	0.047*	
C16	0.4727 (3)	0.1964 (2)	0.5817 (2)	0.0467 (6)	
H16	0.4458	0.1166	0.5302	0.056*	
C17	0.4161 (3)	0.2143 (2)	0.6919 (2)	0.0495 (6)	
H17	0.3520	0.1469	0.7167	0.059*	
C18	0.4562 (3)	0.3343 (2)	0.7653 (2)	0.0419 (5)	
H18	0.4170	0.3465	0.8400	0.050*	
Cl1	1.15819 (9)	0.95211 (6)	1.36830 (5)	0.05451 (17)	
H1WA	0.340 (4)	0.550 (3)	0.984 (2)	0.050 (8)*	
N1	0.7798 (2)	0.62241 (16)	0.69110 (15)	0.0341 (4)	
N2	0.5490 (2)	0.43414 (15)	0.73402 (14)	0.0315 (4)	
N3	0.6788 (2)	0.81919 (15)	0.90636 (14)	0.0315 (4)	
O1	0.4109 (2)	0.69189 (14)	0.73855 (13)	0.0399 (4)	
O2	0.3186 (2)	0.89098 (16)	0.72147 (16)	0.0539 (4)	
O3	0.2802 (2)	0.81929 (16)	0.89951 (15)	0.0504 (4)	
O4	0.7824 (2)	0.59406 (13)	0.96069 (12)	0.0381 (3)	
O1W	0.3995 (2)	0.60387 (17)	0.95374 (16)	0.0495 (4)	
H1WB	0.3365	0.6563	0.9481	0.074*	
S1	0.38106 (7)	0.82061 (5)	0.79767 (5)	0.03552 (13)	

### Atomic displacement parameters (Å^2^)

**Table:**

	*U*^11^	*U*^22^	*U*^33^	*U*^12^	*U*^13^	*U*^23^
Zn1	0.04229 (15)	0.02405 (14)	0.02831 (14)	0.00326 (10)	−0.00157 (10)	−0.00023 (10)
C1	0.0363 (10)	0.0297 (10)	0.0269 (10)	0.0079 (8)	0.0033 (8)	0.0039 (8)
C2	0.0467 (12)	0.0351 (11)	0.0361 (11)	0.0177 (9)	0.0029 (9)	0.0078 (9)
C3	0.0365 (11)	0.0483 (13)	0.0330 (11)	0.0121 (9)	−0.0015 (9)	0.0127 (10)
C4	0.0355 (11)	0.0407 (12)	0.0302 (11)	−0.0001 (9)	−0.0058 (8)	0.0060 (9)
C5	0.0378 (11)	0.0279 (10)	0.0346 (11)	0.0012 (8)	−0.0033 (8)	0.0052 (8)
C6	0.0316 (10)	0.0268 (10)	0.0293 (10)	0.0033 (8)	−0.0009 (8)	0.0044 (8)
C7	0.0344 (10)	0.0221 (9)	0.0320 (10)	0.0023 (8)	−0.0019 (8)	0.0022 (8)
C8	0.0432 (11)	0.0285 (10)	0.0351 (11)	0.0029 (9)	−0.0066 (9)	0.0089 (9)
C9	0.0433 (12)	0.0347 (12)	0.0544 (15)	−0.0009 (10)	0.0061 (11)	0.0053 (10)
C10	0.0452 (13)	0.0442 (14)	0.0691 (17)	0.0052 (11)	0.0157 (12)	0.0223 (13)
C11	0.0506 (14)	0.0620 (16)	0.0459 (14)	0.0147 (12)	0.0137 (11)	0.0255 (12)
C12	0.0427 (12)	0.0491 (13)	0.0316 (11)	0.0126 (10)	−0.0002 (9)	0.0091 (10)
C13	0.0291 (9)	0.0311 (10)	0.0297 (10)	0.0089 (8)	−0.0020 (8)	0.0057 (8)
C14	0.0279 (9)	0.0298 (10)	0.0298 (10)	0.0058 (8)	−0.0046 (8)	0.0022 (8)
C15	0.0435 (12)	0.0357 (12)	0.0330 (11)	0.0064 (9)	−0.0040 (9)	−0.0025 (9)
C16	0.0468 (13)	0.0305 (12)	0.0524 (14)	0.0001 (10)	−0.0051 (11)	−0.0061 (10)
C17	0.0467 (13)	0.0311 (12)	0.0643 (16)	−0.0058 (10)	0.0063 (12)	0.0069 (11)
C18	0.0437 (12)	0.0353 (12)	0.0439 (13)	0.0006 (9)	0.0102 (10)	0.0076 (10)
Cl1	0.0662 (4)	0.0497 (4)	0.0402 (3)	−0.0031 (3)	−0.0221 (3)	0.0057 (3)
N1	0.0362 (9)	0.0283 (9)	0.0346 (9)	0.0031 (7)	0.0010 (7)	0.0028 (7)
N2	0.0331 (8)	0.0270 (9)	0.0313 (9)	0.0035 (7)	0.0022 (7)	0.0019 (7)
N3	0.0369 (9)	0.0246 (8)	0.0321 (9)	0.0045 (7)	−0.0043 (7)	0.0058 (7)
O1	0.0480 (9)	0.0319 (8)	0.0350 (8)	0.0072 (6)	−0.0069 (7)	−0.0015 (6)
O2	0.0584 (10)	0.0472 (10)	0.0587 (11)	0.0110 (8)	−0.0202 (8)	0.0173 (8)
O3	0.0540 (10)	0.0400 (9)	0.0562 (11)	0.0119 (8)	0.0149 (8)	0.0062 (8)
O4	0.0517 (9)	0.0242 (7)	0.0351 (8)	0.0062 (6)	−0.0060 (7)	0.0010 (6)
O1W	0.0585 (11)	0.0410 (10)	0.0561 (11)	0.0136 (8)	0.0200 (9)	0.0211 (8)
S1	0.0397 (3)	0.0292 (3)	0.0357 (3)	0.0067 (2)	−0.0064 (2)	0.0036 (2)

### Geometric parameters (Å, º)

**Table:**

Zn1—O4	1.9851 (15)	C9—C10	1.376 (3)
Zn1—N3	2.0936 (16)	C9—H9	0.9300
Zn1—N2	2.1144 (16)	C10—C11	1.372 (4)
Zn1—N1	2.1817 (18)	C10—H10	0.9300
Zn1—O1W	2.1999 (17)	C11—C12	1.377 (3)
Zn1—O1	2.3507 (15)	C11—H11	0.9300
C1—O4	1.318 (2)	C12—C13	1.383 (3)
C1—C2	1.411 (3)	C12—H12	0.9300
C1—C6	1.420 (3)	C13—N1	1.343 (3)
C2—C3	1.367 (3)	C13—C14	1.482 (3)
C2—H2	0.9300	C14—N2	1.352 (3)
C3—C4	1.388 (3)	C14—C15	1.386 (3)
C3—H3	0.9300	C15—C16	1.379 (3)
C4—C5	1.364 (3)	C15—H15	0.9300
C4—Cl1	1.754 (2)	C16—C17	1.368 (3)
C5—C6	1.414 (3)	C16—H16	0.9300
C5—H5	0.9300	C17—C18	1.378 (3)
C6—C7	1.446 (3)	C17—H17	0.9300
C7—N3	1.287 (2)	C18—N2	1.338 (3)
C7—H7	0.9300	C18—H18	0.9300
C8—N3	1.459 (3)	O1—S1	1.4682 (15)
C8—S1	1.788 (2)	O2—S1	1.4416 (17)
C8—H8A	0.9700	O3—S1	1.4513 (17)
C8—H8B	0.9700	O1W—H1WA	0.84 (3)
C9—N1	1.338 (3)	O1W—H1WB	0.8200
			
O4—Zn1—N3	90.71 (6)	C11—C10—H10	120.9
O4—Zn1—N2	102.79 (6)	C9—C10—H10	120.9
N3—Zn1—N2	164.81 (7)	C10—C11—C12	120.0 (2)
O4—Zn1—N1	104.54 (7)	C10—C11—H11	120.0
N3—Zn1—N1	93.88 (6)	C12—C11—H11	120.0
N2—Zn1—N1	76.17 (6)	C11—C12—C13	118.6 (2)
O4—Zn1—O1W	90.69 (7)	C11—C12—H12	120.7
N3—Zn1—O1W	92.08 (7)	C13—C12—H12	120.7
N2—Zn1—O1W	94.66 (7)	N1—C13—C12	121.86 (19)
N1—Zn1—O1W	163.55 (7)	N1—C13—C14	115.00 (17)
O4—Zn1—O1	165.47 (5)	C12—C13—C14	123.10 (18)
N3—Zn1—O1	78.29 (6)	N2—C14—C15	121.59 (19)
N2—Zn1—O1	89.43 (6)	N2—C14—C13	115.84 (16)
N1—Zn1—O1	85.85 (6)	C15—C14—C13	122.45 (19)
O1W—Zn1—O1	80.36 (6)	C16—C15—C14	119.0 (2)
O4—C1—C2	118.78 (18)	C16—C15—H15	120.5
O4—C1—C6	124.10 (18)	C14—C15—H15	120.5
C2—C1—C6	117.09 (18)	C17—C16—C15	119.6 (2)
C3—C2—C1	122.1 (2)	C17—C16—H16	120.2
C3—C2—H2	118.9	C15—C16—H16	120.2
C1—C2—H2	118.9	C16—C17—C18	118.6 (2)
C2—C3—C4	119.88 (19)	C16—C17—H17	120.7
C2—C3—H3	120.1	C18—C17—H17	120.7
C4—C3—H3	120.1	N2—C18—C17	123.0 (2)
C5—C4—C3	120.61 (19)	N2—C18—H18	118.5
C5—C4—Cl1	119.85 (17)	C17—C18—H18	118.5
C3—C4—Cl1	119.50 (16)	C9—N1—C13	118.41 (19)
C4—C5—C6	120.4 (2)	C9—N1—Zn1	126.34 (15)
C4—C5—H5	119.8	C13—N1—Zn1	113.33 (13)
C6—C5—H5	119.8	C18—N2—C14	118.20 (17)
C5—C6—C1	119.71 (18)	C18—N2—Zn1	125.78 (14)
C5—C6—C7	114.88 (17)	C14—N2—Zn1	116.00 (13)
C1—C6—C7	125.40 (17)	C7—N3—C8	116.93 (17)
N3—C7—C6	126.09 (18)	C7—N3—Zn1	124.61 (14)
N3—C7—H7	117.0	C8—N3—Zn1	118.37 (13)
C6—C7—H7	117.0	S1—O1—Zn1	111.64 (8)
N3—C8—S1	107.87 (14)	C1—O4—Zn1	128.73 (13)
N3—C8—H8A	110.1	Zn1—O1W—H1WA	140.4 (18)
S1—C8—H8A	110.1	Zn1—O1W—H1WB	109.5
N3—C8—H8B	110.1	H1WA—O1W—H1WB	106.3
S1—C8—H8B	110.1	O2—S1—O3	114.48 (11)
H8A—C8—H8B	108.4	O2—S1—O1	113.83 (10)
N1—C9—C10	122.9 (2)	O3—S1—O1	111.93 (10)
N1—C9—H9	118.6	O2—S1—C8	106.08 (10)
C10—C9—H9	118.6	O3—S1—C8	106.82 (10)
C11—C10—C9	118.2 (2)	O1—S1—C8	102.52 (9)
			
O4—C1—C2—C3	−175.1 (2)	C15—C14—N2—C18	1.2 (3)
C6—C1—C2—C3	3.1 (3)	C13—C14—N2—C18	−174.98 (18)
C1—C2—C3—C4	0.9 (3)	C15—C14—N2—Zn1	179.59 (15)
C2—C3—C4—C5	−3.9 (3)	C13—C14—N2—Zn1	3.4 (2)
C2—C3—C4—Cl1	173.90 (17)	O4—Zn1—N2—C18	65.54 (19)
C3—C4—C5—C6	2.6 (3)	N3—Zn1—N2—C18	−142.3 (2)
Cl1—C4—C5—C6	−175.13 (16)	N1—Zn1—N2—C18	167.61 (19)
C4—C5—C6—C1	1.5 (3)	O1W—Zn1—N2—C18	−26.24 (19)
C4—C5—C6—C7	−179.6 (2)	O1—Zn1—N2—C18	−106.52 (18)
O4—C1—C6—C5	173.83 (19)	O4—Zn1—N2—C14	−112.71 (14)
C2—C1—C6—C5	−4.3 (3)	N3—Zn1—N2—C14	39.5 (3)
O4—C1—C6—C7	−5.0 (3)	N1—Zn1—N2—C14	−10.64 (13)
C2—C1—C6—C7	176.93 (19)	O1W—Zn1—N2—C14	155.51 (14)
C5—C6—C7—N3	−178.2 (2)	O1—Zn1—N2—C14	75.23 (14)
C1—C6—C7—N3	0.6 (3)	C6—C7—N3—C8	−178.30 (19)
N1—C9—C10—C11	1.2 (4)	C6—C7—N3—Zn1	5.2 (3)
C9—C10—C11—C12	−3.0 (4)	S1—C8—N3—C7	−140.80 (16)
C10—C11—C12—C13	1.6 (4)	S1—C8—N3—Zn1	35.87 (18)
C11—C12—C13—N1	1.8 (3)	O4—Zn1—N3—C7	−5.63 (17)
C11—C12—C13—C14	−175.8 (2)	N2—Zn1—N3—C7	−158.6 (2)
N1—C13—C14—N2	11.9 (2)	N1—Zn1—N3—C7	−110.26 (17)
C12—C13—C14—N2	−170.33 (19)	O1W—Zn1—N3—C7	85.09 (17)
N1—C13—C14—C15	−164.25 (19)	O1—Zn1—N3—C7	164.80 (18)
C12—C13—C14—C15	13.5 (3)	O4—Zn1—N3—C8	177.97 (15)
N2—C14—C15—C16	−0.7 (3)	N2—Zn1—N3—C8	25.0 (3)
C13—C14—C15—C16	175.22 (19)	N1—Zn1—N3—C8	73.34 (15)
C14—C15—C16—C17	−0.4 (4)	O1W—Zn1—N3—C8	−91.31 (15)
C15—C16—C17—C18	0.9 (4)	O1—Zn1—N3—C8	−11.60 (14)
C16—C17—C18—N2	−0.4 (4)	O4—Zn1—O1—S1	20.4 (3)
C10—C9—N1—C13	2.1 (3)	N3—Zn1—O1—S1	−21.04 (9)
C10—C9—N1—Zn1	−161.05 (18)	N2—Zn1—O1—S1	167.95 (10)
C12—C13—N1—C9	−3.6 (3)	N1—Zn1—O1—S1	−115.88 (10)
C14—C13—N1—C9	174.19 (19)	O1W—Zn1—O1—S1	73.13 (10)
C12—C13—N1—Zn1	161.63 (16)	C2—C1—O4—Zn1	−179.41 (14)
C14—C13—N1—Zn1	−20.6 (2)	C6—C1—O4—Zn1	2.5 (3)
O4—Zn1—N1—C9	−79.2 (2)	N3—Zn1—O4—C1	1.91 (17)
N3—Zn1—N1—C9	12.5 (2)	N2—Zn1—O4—C1	174.89 (17)
N2—Zn1—N1—C9	−179.1 (2)	N1—Zn1—O4—C1	96.10 (17)
O1W—Zn1—N1—C9	123.5 (2)	O1W—Zn1—O4—C1	−90.18 (17)
O1—Zn1—N1—C9	90.44 (19)	O1—Zn1—O4—C1	−38.5 (3)
O4—Zn1—N1—C13	116.92 (14)	Zn1—O1—S1—O2	154.40 (10)
N3—Zn1—N1—C13	−151.34 (14)	Zn1—O1—S1—O3	−73.84 (11)
N2—Zn1—N1—C13	17.03 (13)	Zn1—O1—S1—C8	40.30 (11)
O1W—Zn1—N1—C13	−40.4 (3)	N3—C8—S1—O2	−168.93 (14)
O1—Zn1—N1—C13	−73.40 (14)	N3—C8—S1—O3	68.55 (16)
C17—C18—N2—C14	−0.6 (3)	N3—C8—S1—O1	−49.28 (16)
C17—C18—N2—Zn1	−178.84 (18)		

### Hydrogen-bond geometry (Å, º)

**Table:**

*D*—H···*A*	*D*—H	H···*A*	*D*···*A*	*D*—H···*A*
O1*W*—H1*WA*···O4^i^	0.84 (3)	1.96 (3)	2.791 (2)	172 (3)
O1*W*—H1*WB*···O3	0.82	2.10	2.855 (2)	154

Symmetry code: (i) −*x*+1, −*y*+1, −*z*+2.
